# Up-regulated miR155 Reverses the Epithelial-mesenchymal Transition Induced by EGF and Increases Chemo-sensitivity to Cisplatin in Human Caski Cervical Cancer Cells

**DOI:** 10.1371/journal.pone.0052310

**Published:** 2012-12-20

**Authors:** Cui Lei, Yanlin Wang, Yurong Huang, Han Yu, Yiling Huang, Liting Wu, Liming Huang

**Affiliations:** 1 Department of Oncology, Zhongnan Hospital, Wuhan University, Wuhan, People’s Republic of China; 2 Institute of Molecular Biology of Three Gorges University, Yichang, People’s Republic of China; 3 Department of Gynecology, Renhe Hospital, Three Gorges University, Yichang, People’s Republic of China; Wayne State University School of Medicine, United States of America

## Abstract

The epithelial-mesenchymal transition (EMT) induced by EGF promotes cervical cancer progression; however, the mechanisms underlying the EGF-induced EMT remain unclear. In this study, we reported that miR155 overexpression suppressed EGF-induced EMT, decreased migration/invasion capacities, inhibited cell proliferation and increased the chemo-sensitivity to DDP in human Caski cervical cancer cells. Further, the overexpression of miR155 increased TP53 expression but reduced SMAD2, and CCND1 expression levels. These data suggest that miR155 negatively regulates EGF-induced EMT. We conclude that miR155 does not act as an oncogene but as a tumour suppressor in Caski cells.

## Introduction

Cervical cancer is the second largest class of malignant tumours for women, and it endangers women's health, especially in developing countries. Metastasis and invasion are the main reasons for death in cervical cancer cases, thus it is important to clarify the molecular mechanisms of these phenomena. It has been reported that the epithelial to mesenchymal transition (EMT) is an important process involved in tumour metastasis and invasion [1]. The main features of EMT include the dissolution of epithelial tight junctions, remodelling of the cytoskeleton, the loss of apical-basal polarity, and the acquisition of mesenchymal markers, such as N-cadherin and vimentin. EMT endows tumour cells with higher invasive/metastatic capacities, stem cell-like characteristics, resistance to apoptosis, and immune tolerance [2].

EGF (Epithelial growth factor) is one of the most important EMT regulatory factors that triggers EMT in a variety of solid tumours, including cervical cancer. It has been reported that the tumours with high EGF receptor expression have poor clinical prognosis, and EGF-induced EMT may be one reason for this [3]–[5]. Thus, preventing EGF-induced EMT could be an appropriate method to inhibit invasion and metastasis.

Recent studies have suggested that miRNAs play an important role in the regulation of EMT [6], [7]. miRNAs are 18- to 25-nucleotide-long noncoding RNAs that can regulate gene expression by accelerating the degradation and inhibiting the translation of target mRNAs. Among the miRNAs identified to date, miR155 is associated with tumor proliferation and is overexpressed in many human tumours [8]. One study illustrated that the abnormal expression of miR155 was an early event in pancreatic cancer and closely related to a low survival rate [9]. In endometrial cancer, the occurrence of EMT was accompanied by elevated miR155 expression levels [10]. It is not yet clear whether miR155 is involved with the occurrence of EMT in cervical cancer. In this study, using EGF as an EMT-inducing factor in human cervical cancer cells, we explored the regulatory roles of miR155 in the EMT process, cellular proliferation, cellular sensitivity to chemotherapeutic drugs, and evaluated the potential value of miR155 as a molecular target for the early prevention of cervical cancer invasion and metastasis.

## Materials and Methods

### Cell Lines

Caski cells was purchased from the Cell Bank of China (Wuhan) and were cultured at 37°C in 5% CO2 in RPMI-1640 containing 10% foetal bovine serum (FBS), 100 µg/ml streptomycin, and 100 units/ml penicillin.

### RNA Isolation and miRNA Detection

RNA from the cultured cells was isolated with Trizol reagent (Invitrogen) and was then used to synthesise first strand cDNA. Detection of the matured miRNAs was performed with PCR using the SYBR Premix Ex Taq ^tm^ (TAKARA). U6 was used as an internal control. The primers used in this experiment are shown in Table S1.

### Plasmid Construction and Stable/transient Transfection of miR155

A human genomic DNA fragment of approximately 400 bp containing the miR155 sequence was cloned into the pcDNA3.1-GFP vector. The resulting plasmid pcDNA3.1-GFP-miRNA-155 carries a recombinant DNA sequence for GFP and the miR155-containing fragment. To generate a cell line that stably expresses miR155, Caski cells were transfected with pcDNA3.1-GFP-miR155 using Lipofectamine 2000 reagent (Invitrogen). Following selection with G418, the single clone that over-expressed miR155 was identified. For miR155 transient overexpression, miR155 mimics (RIBOBRO) were used to transfect the Caski cells.

### *In vitro* Migration and Invasion Assays

A Matrigel-based transwell assay was used to assay cell migration and invasion *in vitro* as described previously [11]. For analysis of the invasive properties, 2×10^4^ cells were seeded on top of the Matrigel-coated cell culture inserts in 200 µl RPMI-1640 medium without FBS and incubated for 24 hours. The inserts were then washed with phosphate buffered saline (PBS) and fixed in 4% paraformaldehyde. After being stained with haematin, the invasive cells were counted under the microscope. The migration assay was performed by the same way described above except that Matrigel was not coated into the inserts.

### Western Blot (WB)

Total protein was extracted from cells using cell lysis buffer (0.5% NP-40, 0.5% SDS, 1.5 mM Tris-HCl pH 7.4, and 15 mM NaCl). Protein samples (20 ug/lane) were electrophoresed, transferred to PVDF membranes and incubated overnight with primary antibodies against E-cadherin (sc-7870, Santa Cruz Biotechnology), N-cadherin (BA0637, BOSTER), MMP1 (130-1-16, RayBiotech), and Annexin A2 (A2485, Sigma). The membranes were treated with goat-anti-rabbit HRP-conjugated secondary antibodies (Invitrogen). The target protein bands were determined using the reagents provided in the ECL+plus kit (GE healthcare, Piscataway, NJ, USA).

### Immunoﬂuorescence Analysis

Immunoﬂuorescence analysis was used to assay the expression levels of E-cadherin with antibodies against E-cadherin (sc-7558, Santa Cruz Biotechnology), and the expression of N-cadherin with antibodies against N-cadherin (BA0637, BOSTER) as described previously [12].

### Cell Proliferation Assay

Caski and caski-miR155 were seeded into 96-well culture plates and grown to 70% confluence. The cells were treated with DDP (cis-diamminedichloroplatinum, DDP) for 0–3 days in RPMI-1640 medium. The medium in each well was removed, and 100 µl (methyl thiazolyl tetrazolium, MTT) solution (0.25 g/L in RPMI-1640 medium) was added. After incubation at 37°C for 4 hours, the medium was removed and 200 µl DMSO was added to each well. Absorbance (A) at 570 nm was recorded. The cell growth rate and inhibitor rate were calculated as follows:
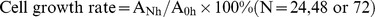


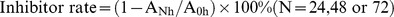



### Flow Cytometry

Caski and Caski-miR155 cells were harvested in the logarithmic growth phase and fixed overnight with 80% ethanol. The cells were then washed with cold PBS, stained with propidium iodide (PI, 0.05 mg/ml) and RNase A (0.5 mg/ml). Flow cytometry analysis was used to determine the distribution of cells in cell cycle sub-phases and to measure the apoptosis peak induced by DDP (10 µmol/ml) for 24 hours.

### Statistical Analysis

All data were presented as the mean ± standard deviation (SD). Statistical analysis between groups was assessed by Student’s two-tailed t-test and ANOVA. P-values less than 0.05 were regarded as statistically significant.

## Results

### EGF Induces EMT in Caski Cells

**Figure 1:**
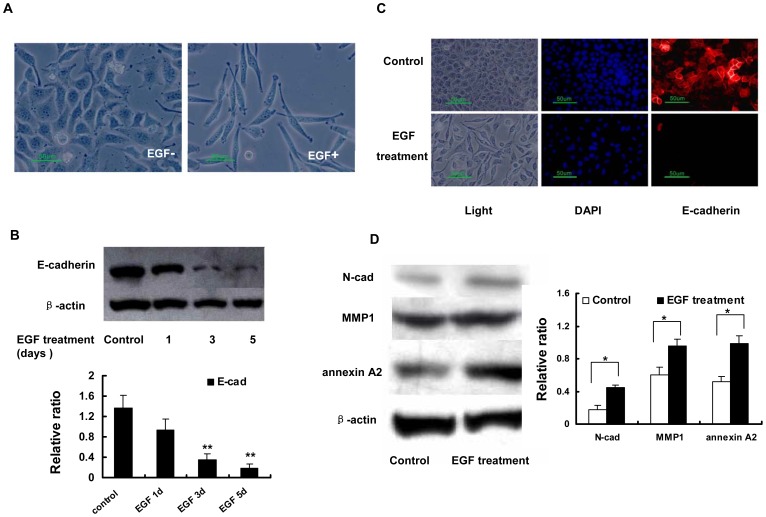
EGF exposure induces EMT in Caski cells. Caski cells were cultured under routine conditions with or without EGF (50 ng/ml) for 1 to 5 days. A. The morphological changes caused by EGF were observed by microscopy. B. Downregulation of E-cadherin by EGF treatment was determined by western blot. Densitometric analysis of three independent triplicate Western blots. C. E-cadherin downregulation in response to EGF (50 ng/ml) treatment for 3 days was verified by IF. D. Upregulation of N-cad, MMP1 and annexin A2 in the EMT process as investigated by western blot. Densitometric analysis of three independent triplicate Western blots. Values are the average of triple determinations with the S.D. indicated by error bars. *P<0.05, ** P<0.01, compared to control.

It has been reported that the EGF receptor is highly elevated in the cervical cancer cells, suggesting that EGF exposure may induce EMT in the cervical cell lines [13], [14], [15]. In this experiment, Caski cells were cultured in routine conditions with or without EGF (50 ng/mL) for 1, 3, or 5 days, and the EMT-related changes were determined. The morphological change was first visualised in this process. After EGF stimulation, Caski cells became more fusiform and the connection between the cells decreased (Figure 1A). When E-cadherin (E-cad) expression was assayed by WB, the results illustrated that EGF exposure downregulated E-cad expression compared to the control cells (Figure 1B). The similar downregulation of E-cad expression was verified by an IF assay in which Caski cells were treated with EGF (50 ng/ml) for 3 days (Figure 1C). At the same time point, we also detected the expression of other EMT-related factors (N-cadherin, MMP1 and annexin A2) by WB and found that all three factors were upregulated (Figure 1D). These data indicate that EGF can induce EMT in the Caski cells.

### miR155 is Upregulated in the EGF-induced EMT

**Figure 2:**
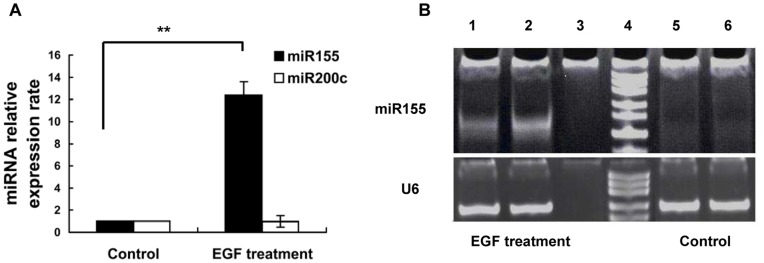
Effects of EGF on miRNA levels of miR155 and miR200c in Caski Cells. A. The relative expression levels of miR155 and miR200c were detected by real-time PCR. miR155 was upregulated with 3 days of EGF treatment (50 ng/ml), and no change was found in miR200c expression level under EGF stimulation. The relative miR155 expression ratio in EGF treatment/Control is 12.21. Experiments were repeated 3 times. T-test was used for statistical analysis; ** P<0.01. B. The upregulated miR155 expression level was verified by reverse-transcription PCR. U6 was used as an internal control, Lane 1 and 2 are EGF treatment, lane 3 is negative control, 4 lane is marker, lane 5 and 6 are control (without EGF).

Total RNA was purified from the EGF-induced mesenchymal-like Caski cells and normal Caski cells. The expression levels of miR155 or miR200c were detected by performing real-time PCR on these total RNAs. The results showed that compared to the normal Caski cells, miR155 was greatly upregulated in the mesenchymal Caski cells, but no change of expression level was observed for miR200c, which was found to be downregulated during the EMT process in many solid tumours by other studies [16], [17] (Figure 2).

### The Overexpression of miR155 Inhibits EMT

**Figure 3:**
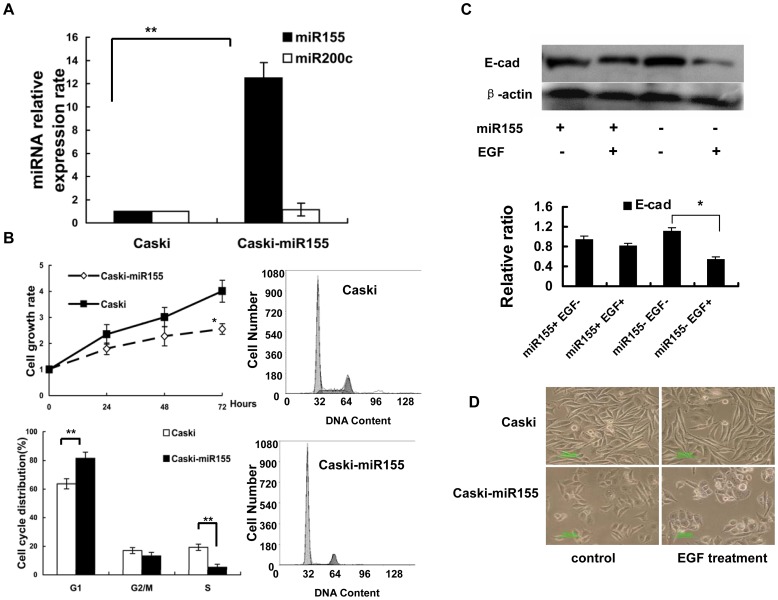
miR155 overexpression inhibits EMT. A. After pcDNA3.1-GFP-miRNA-155 transfection, the miR155 expression level increased more than 12-fold, but no significant difference was observed for miR200c expression. The experiment was repeated independently 3 times. **P<0.01 compared to the control cells. B. Cell proliferation was tested by MTT. The results show that miR155 overexpression significantly inhibited the cell growth rate. P<0.05 compared to the control cells. Flow cytometry was used to test effects of miR155 overexpression on the cell cycle. The results show a decreased cell ratio in S phase (17% in Caski control cells, 5.4% in Caski-miR155) but an increased cell ratio in G1 phase (63.7% in Caski control cells, 81.4% in Caski-miR155). The experiment was repeated 3 times independently. P<0.01 compared to the control cells. C. E-cad expression was tested by western blot. E-cadherin expression level was greatly decreased by 3 days of EGF (50 ng/ml) exposure in Caski cells. Densitometric analysis of three independent triplicate Western blots. Values are the average of triple determinations with the S.D. indicated by error bars. *P<0.05. This effect was partially prevented by transfection with miR155 mimics. D. The morphological changes caused by miR155 overexpression. Caski-miR155 cells are more cubical than normal Caski cells. When treated with EGF (50 ng/ml) for 3 days, only a small fraction of Caski-miR155 cells were transformed into the fusiform-like cells.

Although miR155 was greatly upregulated in the EGF-induced EMT process, it is possible that this is only a compensatory event or an unrelated phenomenon. To further clarify the role of miRNA155 in the EMT process, a Caski cell line with over-expressed miR155 (Caski-miR155) was created by transfecting pcDNA3.1-GFP-miR155 plasmid into the Caski cells, with miR155 expression being verified by real-time PCR (Figure 3A). Using this cell line as a model, we found that the overexpression of miR155 inhibited cell growth and interfered with the cell cycle by decreasing the cell ratio in the S phase (17% in Caski control cells, 5.4% in Caski-miR155) but increasing the cell ratio in the G1 phase (63.7% in Caski control cells, 81.4% in Caski-miR155), as shown in Figure 3B. The EGF-induced downregulation of E-cadherin expression was partly reversed by transiently transfected miR155 mimics (Figure 3C). With miR155 overexpression, the morphology of the cells became more cuboidal. After 3 days of treatment with EGF (50 ng/ml), only a small portion of Caski-miR155 cells were transformed to fusiform cells (Figure 3D). These results indicate that the overexpression of miR155 inhibited EGF-induced EMT.

**Figure 4:**
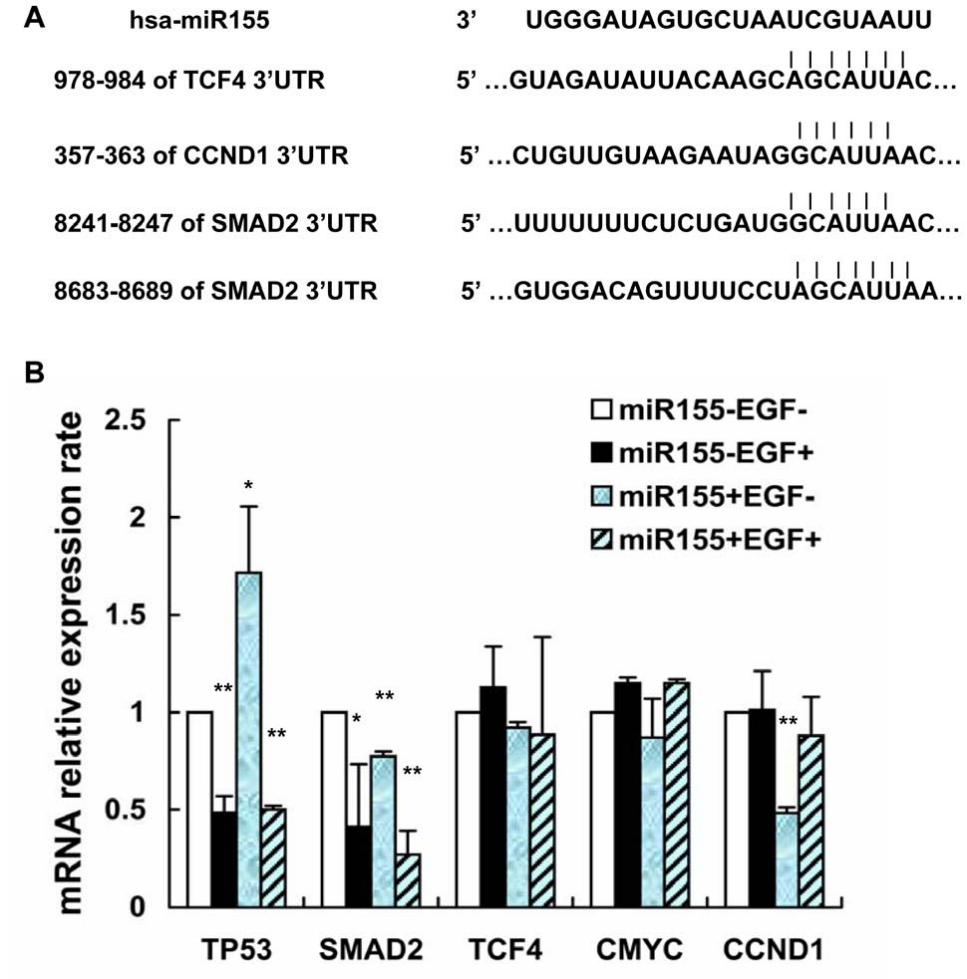
Effect of miR155 on the expression of target genes. A. Predicted miR155-binding sites in the 3′UTR of SMAD2, TCF4 and CCND1 mRNAs. B. TP53, SMAD2, TCF4, CMYC and CCND1 mRNA expression levels were assayed by real-time PCR in Caski cells treated with EGF (50 ng/ml) for 3 days, with or without transfection of miR155 mimics. TP53 was downregulated by EGF treatment but significantly upregulated by miR155 overexpression. This increased TP53 expression was reversed by EGF treatment. SMAD2 was downregulated by both EGF treatment and miR155 overexpression. Treating miR155-overexpressing cells with EGF resulted in a further decrease of SMAD2. No significant changes in TCF4 or CMYC mRNA were observed between EGF-treated and miR155 mimic-transfected cells. CCND1 mRNA expression was downregulated by miR155 overexpression, and this downregulation was partially reversed by EGF treatment.

TP53 and SMAD2 have been linked to the EMT regulatory pathway [18], [19]. By screening Targetscan (http://www.targetscan.org), TCF4, CCND1 and SMAD2 were predicted to be the target genes of miR155. Thus, we investigated TP53, SMAD2, TCF4, CMYC and CCND1 mRNA expression levels in Caski cells treated with EGF (50 ng/ml) for 3 days with or without transfection of miR155 mimics (Figure 4). The results showed that TP53 was downregulated in the EGF-induced EMT process and upregulated by miR155 overexpression, but the TP53 expression induced by miR155 was thoroughly reversed by EGF treatment. miR155 overexpression decreased SMAD2 expression. Although TCF4 was verified as a miR155 target protein by another group [20], no statistically significant changes were observed in our experiments for TCF4 mRNA expression in EGF-induced and miR155 mimic transfected cells. Additionally, no changes were observed for CMYC, which is a downstream target of and is regulated by TCF4. CCND1 (a cell cycle regulatory protein and another downstream target of TCF4) mRNA expression level was downregulated by miR155 overexpression and can be partly reversed by EGF treatment. Thus, we supposed that miR155 reversed EGF-induced EMT by upregulating TP53, downregulating SMAD2 expression levels, and restraining cell growth by inhibiting CCND1 expression.

### The Overexpression of miR155 Inhibits the Migration and Invasion Abilities of Caski Cells

**Figure 5:**
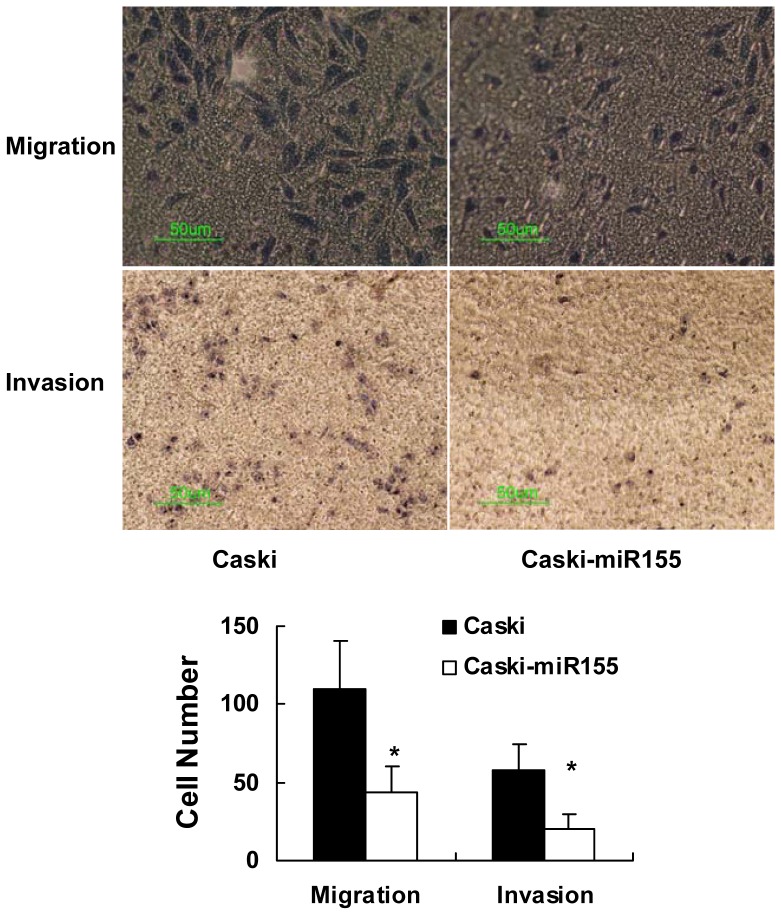
Migration and invasion assay. Caski-miR155 and Caski cells were seeded in Transwell inserts with or without Matrigel. The inserts were fixed and stained after 24 hours, and then the migratory/invading cells were counted under a microscope. The number of Caski-miR155 cells passing through the membrane was significantly lower than that in the normal Caski cells, whether detected by migration or invasion assays. Values are the average of triple determinations with the S.D. indicated by error bars. *P<0.05.

To elucidate the effects of miR155 on migration and invasion in Caski cells *in vitro*, transwell invasion/migration assays were performed (Figure 5). Caski-miR155 and normal Caski cells were seeded in the inserts. After 24 hours, the number of the cells that had transferred to the lower side of the membrane was calculated to quantify the invasion and migration capacities. The number of cells that passed through the membrane was significantly lower in the Caski-miR155 cells than in the normal Caski cells. Thus, we supposed that miR155 prevents the invasive and migration abilities in Caski cells *in vitro*.

### miR155 Increased the Chemo-sensitivity of Caski Cells to DDP

**Figure 6:**
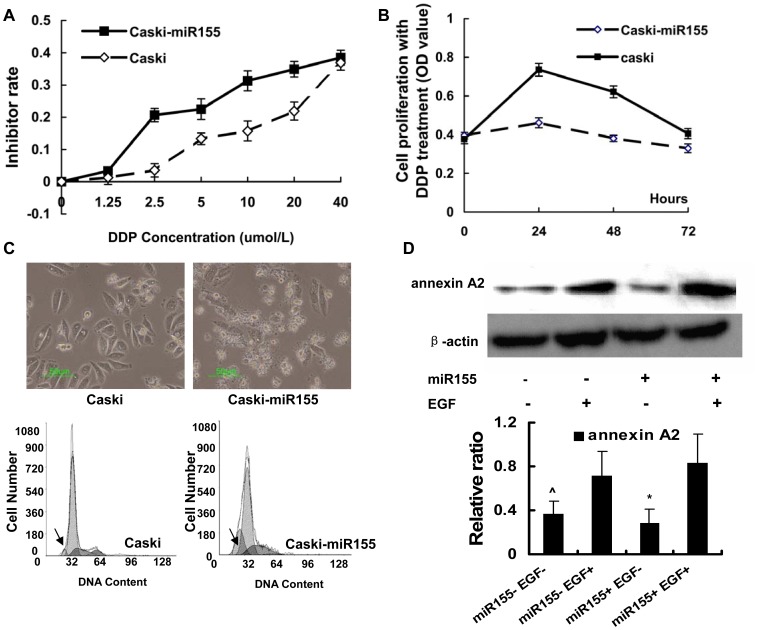
miR155 overexpression increased the chemo-sensitivity of Caski cells to DDP. A. Caski and Caski-miR155 cells were treated with different doses of DDP for 24 hours, and the rate of inhibition was evaluated by an MTT assay. Mean ± SD, n = 4, P<0.05 by ANOVA. B. Cell proliferation was evaluated with an MTT assay. Caski and Caski-miR155 cells were treated with DDP (10 µmol/L) for 0–3 days. Mean ± SD, n = 4, P<0.05 by ANOVA. C. Microscopy was used to observe apoptosis of Caski and Caski-miR155 cells induced by DDP (10 µmol/L) for 1 day. Cells were harvested, and FCM was used to assay apoptosis. The arrows indicate the location of the apoptosis peak. D. Annexin A2 expression was determined by western blot. The Annexin A2 expression was not regulated by miR155 overexpression and could be upregulated by EGF treatment. Densitometric analysis of three independent triplicate Western blots.ˆP>0.05, miR155- compared to miR155+, *P<0.05, EGF- compared to EGF +.

The MTT method was used to detect the effect of miR155 overexpression on the chemo-sensitivity of Caski cells to DDP. The results indicated that the inhibition rate of Caski-miR155 when treated by DDP were much higher than that of the normal Caski cells in a dose-dependent manner. The proliferation rate of Caski-miR155 cells when treated by DDP was much slower than that of the normal Caski cells in a time-dependent manner (Figure 6 A, B). Treatment with DDP (10 µmol/L) for 24 hours resulted in a much higher apoptotic cell ratio in the Caski-miR155 cells than in the normal Caski cells (Figure 6C). The expression level of annexin A2, a protein involved with apoptosis resistance, was determined by WB. These results did not show that annexin A2 was downregulated by miR155 overexpression (Figure 6D).

## Discussion

EMT is a dynamic process that can be regulated and reversed by many factors, including miRNAs. In this study, we used the human Caski cervical cancer cell line as the model to investigate the role of miR155 in EGF-induced EMT and tried to answer the question of whether miR155 regulates EMT and has a role in metastasis and chemo-sensitivity.

We first used EGF to induce EMT in the Caski cells, which was verified by E-cadherin, N-cadherin, expression and cell morphology. The miR155 expression level was determined by RT-PCR. A 12-fold increase of miR155 expression level was observed following EGF treatment, but no change was found for miR200c, which had been reported to be downregulated in pancreatic cancer and other solid tumours [16], [17]. Thus we concluded that miR155, but not miR200c, is involved in EGF-induced EMT in Caski cells. Similar data published by another group also indicate that miR155 is upregulated in EMT observed in endometrial carcinosarcoma [10].

To elucidate the role of miR155 in EMT, Caski cell lines overexpressing miR155 overexpression were constructed by stable and transient transfections. Interestingly, aberrant overexpression of miR155 dramatically inhibited cell proliferation, inhibited migration/invasion, and promoted the chemo-sensitivity of Caski cells to DDP *in vitro*. At the same time, we found that the cells overexpressing miR155 became more cubical, and only a small portion of the cells had transformed into fusiform cells after 3 days of EGF treatment. The downregulation of E-cadherin caused by EGF treatment was also partly reversed by miR155 mimics in Caski cells.

Previous research had suggested that miR-155 was an onco-miR and that it overexpressed in a number of human malignancies, including B-cell lymphoma and carcinomas of the breast, colon, lung, and ovary [8], [9]. It has been reported that miR-155 repressed P53-induced nuclear protein 1 (TP53NP1) and led to pancreatic tumour development [21]. In breast cancer, miR155 enhanced the JAK2/STATS signalling pathway and transfection of miR155 mimics promoted MDA-MB-231 and MCF-7 cell proliferation [22]. Our data, however, indicate that miR155 overexpression inhibits cell proliferation in Caski cells and prevents EMT. Similar attitude on miR155 function was approved by another group [20], they proved that miR155 prevented EMT by decreasing TCF-4 expression level in 4T1 breast tumor cells.

**Figure 7:**
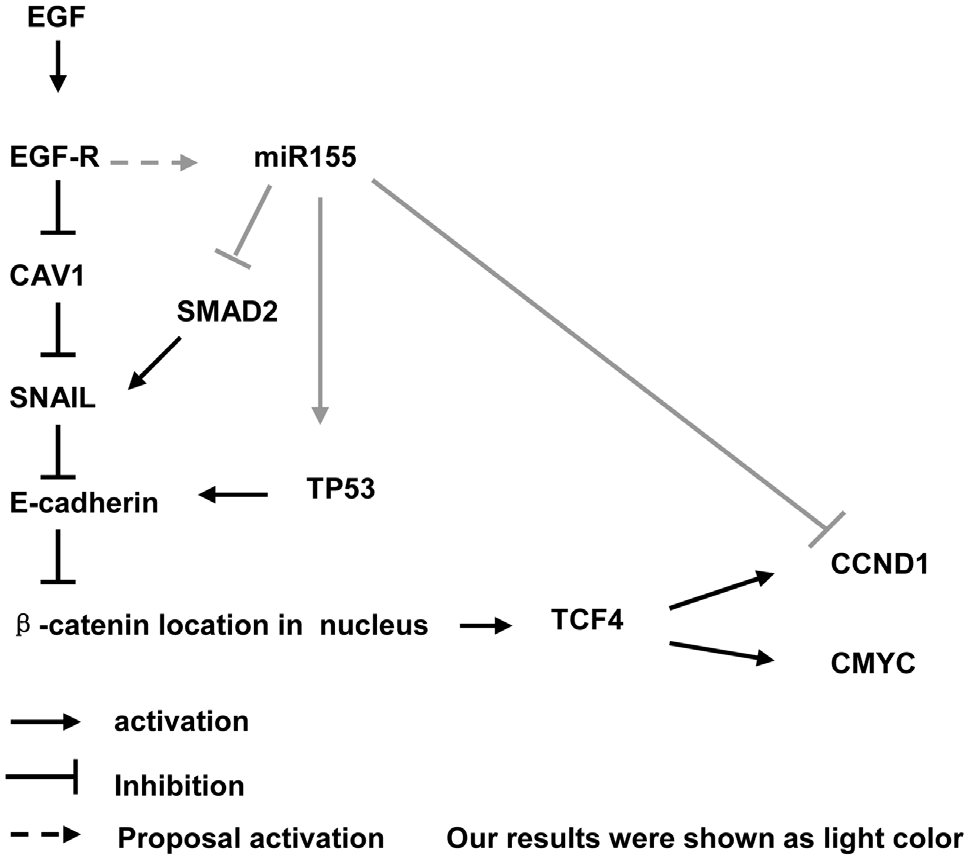
The signal pathway related with EGF-induced EMT.

To elucidate the exact mechanism of miR155 in cell proliferation and EMT in Caski cells, we assayed the expression levels of some factors related to cell growth and EMT (Figure 7). The results showed that TP53 expression was upregulated by miR155 overexpression and downregulated by EGF. Interestingly, EGF-induced E-cadherin downregulation was thoroughly reversed by transfection with miR155 mimics. Recent study suggested that wild TP53 controlled the efficiency by which mammary epithelial cells undergo EMT in response to TGFβ [23]. Thus we supposed miR155 reversed EMT through upregulating TP53 expression.

The SMAD2 signalling pathway is involved in the regulation of proliferation, differentiation, apoptosis and EMT [23]. In this study, we found that, with miR155 overexpression, SMAD2 expression was downregulated. Because there are two miR155 binding sites within the 3′UTR of the SMAD2 mRNA, we suggest that miR155 negatively regulates EMT through the inhibition of Smad2 expression.

Our results indicate that miR155 suppressed the migration and invasion abilities of Caski cells *in* *vitro*. A similar result has been reported in another study with 4T1 breast tumour cells, in which miR155 was found to directly suppress the expression of TCF4 and negatively regulate EMT [20]. In our study, no statistically significant changes in TCF4 expression level were observed in the Caski cells treated with or without EGF and miR155 mimics. Recent study suggested that CCND1 increased the migratory ability and cause EMT in breast cancer [24]. CMYC and CCND1 are downstream genes regulated by TCF4 transcription factor. Unlike CMYC, there is a miR155-binding site in the CCND1 3′UTR. Consistent with this finding, miR155 overexpression obviously downregulated CCND1 level but had no effect on CMYC expression. This finding indicated that, in addition to the TCF4 pathway, CCND1 might also be directly regulated by miR155.

Annexin A2 was considered to be a potential factor for the regulation of cell growth, invasion and chemo-resistance [25]. Our data showed that EGF treatment lead to a increase in Annexin A2 expression. And there is no change in Annexin A2 expression under miR155 overexpression. Further studies are needed to clarify the mechanism of Annexin A2 regulation by EGF.

In summary, we have demonstrated that, in Caski cells, miR155 did not act as an oncogene but as a tumour suppressor. miR155 negatively regulated EGF-induced EMT, decreased proliferation, inhibited migration/invasion and increased chemo-sensitivity in Caski cells in vitro. In EGF-induced EMT, the upregulation of miR155 is an event that cells can compensate for. Our study shows a new aspect of miR155 and its roles in tumour proliferation and metastasis in cervical cancer.

## Supporting Information

Table S1 **Primers for Real-time PCR.**
(TIF)
