# Supplementary figures and images for: Up-regulated miR155 Reverses the Epithelial-mesenchymal Transition Induced by EGF and Increases Chemo-sensitivity to Cisplatin in Human Caski Cervical Cancer Cells
Source: PLoS One. 2012 Dec 20;7(12):e52310. doi: 10.1371/journal.pone.0052310 (PMC3527539; doi:10.1371/journal.pone.0052310)

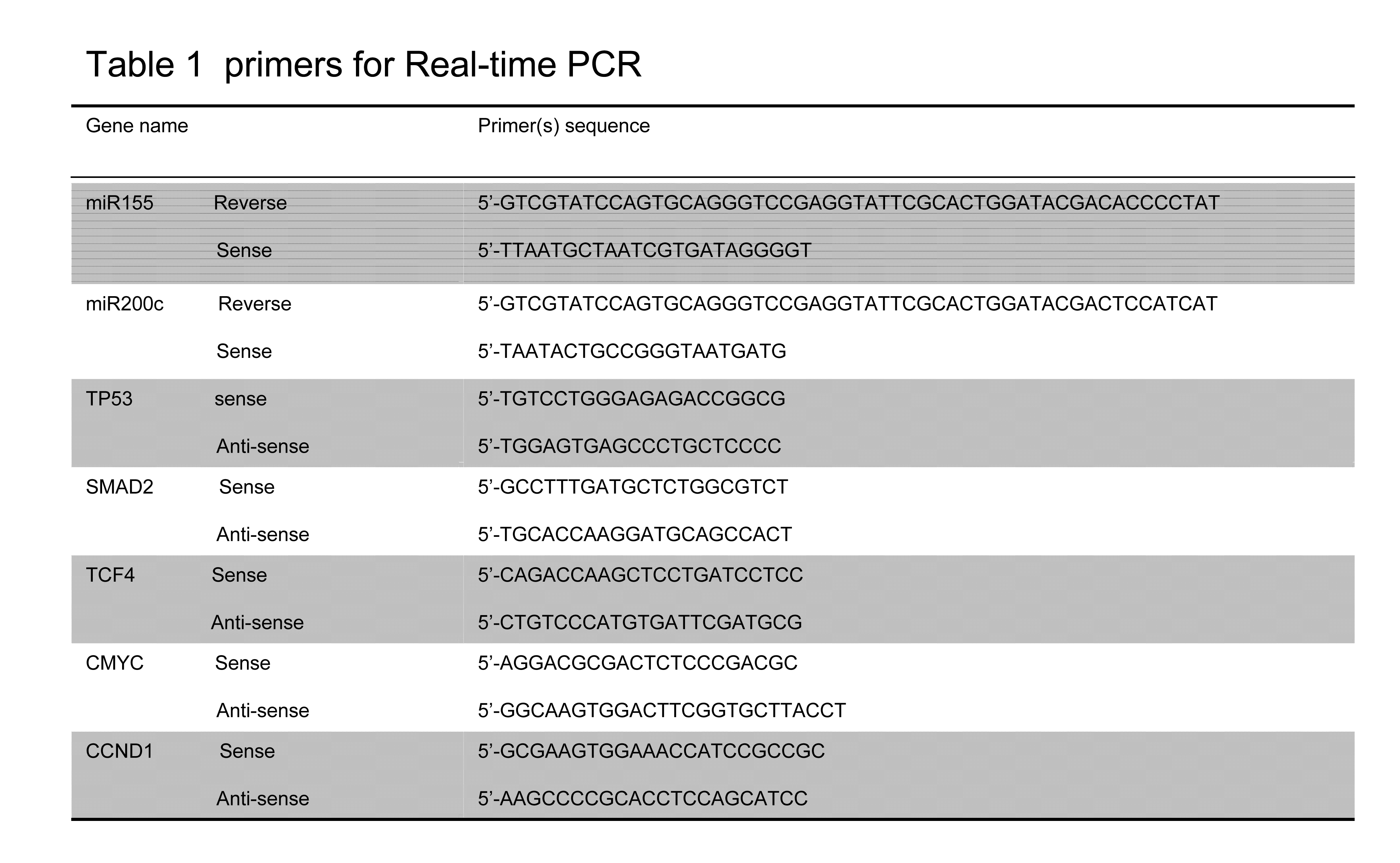

Supplement: Table S1 — Primers for Real-time PCR. (TIF) [file pone.0052310.s001.tif]
